# A Detection Method for Crop Fungal Spores Based on Microfluidic Separation Enrichment and AC Impedance Characteristics

**DOI:** 10.3390/jof8111168

**Published:** 2022-11-05

**Authors:** Xiaodong Zhang, Boxue Guo, Yafei Wang, Lian Hu, Ning Yang, Hanping Mao

**Affiliations:** 1 School of Agricultural Engineering, Jiangsu University, Zhenjiang 212013, China; 2 Key Laboratory of Modern Agricultural Equipment and Technology, Ministry of Education, Jiangsu University, Zhenjiang 212013, China; 3 Key Laboratory of Key Technology on Agricultural Machine and Equipment, Ministry of Education, South China Agricultural University, Guangzhou 510642, China; 4 School of Electrical and Information Engineering, Jiangsu University, Zhenjiang 212013, China

**Keywords:** air-borne diseases, microfluidic chips, fungal spores, impedance characteristics

## Abstract

The timely monitoring of airborne crop fungal spores is important for maintaining food security. In this study, a method based on microfluidic separation and enrichment and AC impedance characteristics was proposed to detect spores of fungal pathogens that cause diseases on crops. Firstly, a microfluidic chip with tertiary structure was designed for the direct separation and enrichment of *Ustilaginoidea virens* spores, *Magnaporthe grisea* spores, and *Aspergillus niger* spores from the air. Then, the impedance characteristics of fungal spores were measured by impedance analyzer in the enrichment area of a microfluidic chip. The impedance characteristics of fungal spores were analyzed, and four impedance characteristics were extracted: absolute value of impedance (abs), real part of impedance (real), imaginary part of impedance (imag), and impedance phase (phase). Finally, based on the impedance characteristics of extracted fungal spores, K-proximity (KNN), random forest (RF), and support vector machine (SVM) classification models were established to classify the three fungal spores. The results showed that the microfluidic chip designed in this study could well collect the spores of three fungal diseases, and the collection rate was up to 97. The average accuracy of KNN model, RF model, and SVM model for the detection of three disease spores was 93.33, 96.44 and 97.78, respectively. The F1-Score of KNN model, RF model, and SVM model was 90, 94.65, and 96.18, respectively. The accuracy, precision, recall, and F1-Score of the SVM model were all the highest, at 97.78, 96.67, 96.69, and 96.18, respectively. Therefore, the detection method of crop fungal spores based on microfluidic separation, enrichment, and impedance characteristics proposed in this study can be used for the detection of airborne crop fungal spores, providing a basis for the subsequent detection of crop fungal spores.

## 1. Introduction

Airborne fungal diseases of crops are difficult to control due to their strong spreading ability, long survival time, and great impact on crops [1]. Once they occur, they can easily cause agricultural economic losses and affect food security. Airborne fungal diseases belong to bioaerosols, and spores are the main propagation mode. Fungal spores in the air are easily dispersed by wind, rain, and air [2], and then germinate rapidly under appropriate temperature and humidity, resulting in the outbreak of plant diseases [3]. At the initial stage of disease outbreak, we can detect the spores and formulate scientific prevention and control suggestions according to the detection results. Therefore, how to quickly and accurately detect airborne fungal spores and take effective control measures in a timely manner is of great significance for maintaining food security and promoting the development of agricultural economy.

At present, the traditional crop disease detection methods mainly include spectral detection, PCR analysis, image recognition and so on. Spectral detection has been widely used in the detection of fungi. The spectral detection method can accurately detect pathogenic bacteria after culture [4,5,6]. For example, Huang et al. [7] used hyperspectral to detect the spectral data of rice panicle blast, and it could accurately grade rice panicle blast. Rice panicle blast grading is very important in gauging cultivar resistance and in the precise control of a blast epidemic. However, this method requires picking infected rice and conducting experiments in the laboratory, which increases the detection time, has a certain lag in disease prevention and control, and increases the complexity of the operation, making it easy to miss the optimal window period for disease prevention and control. PCR analysis is a standard method for detecting microorganisms, which has high sensitivity and accuracy [8,9,10]. For example, Kishore et al. [11] established a dual qPCR method for the simultaneous detection of powdery mildew and downy mildew. While the speed of PCR analysis has improved dramatically, PCR techniques often require sample preparation. Complex sample PCR preparation is a major obstacle to rapid detection of spores, and PCR detection usually takes an hour or more. PCR analysis requires laboratory facilities, expensive equipment, and skilled personnel.

In recent years, impedance detection has been widely used in the detection of microorganisms such as bacteria, spores, cancer cells and PM2.5 [12,13,14]. For example, Subhan et al. [15] used electrochemical impedance spectroscopy combined with microfluidic chip to monitor the growth of microbial cells, and the results showed that this method was particularly suitable for the early detection of bacterial contamination. Pedro et al. [16] proposed a device consisting of a microfluidic chip and an electrochemical impedance spectroscopy, which was able to detect airborne sclerotinia stem rot of canola, which could be used for airborne spore detection and disease prevention. The impedance detection method is easy to operate, easy to carry, low-cost, high-precision, and has low requirements on the operating environment. Therefore, the impedance detection method is expected to become a label-free, non-invasive, and high precision detection method for fungal spores. However, airborne microorganisms are extremely small and complex, and ensuring the accuracy of detection requires enrichment of fungal spores from airborne microorganisms. Traditional spore sampling equipment, such as spore sampler, spore capture instrument, etc., is large and difficult to carry, and the concentration of collected fungal spores is too low, so it is usually impossible to directly detect [17]. The capture and detection of microorganisms in the air is developing towards miniaturization, intelligence and integration [18]. Microfluidic chips have the characteristics of high classification efficiency, simple structure, miniaturization, and low cost [19], and they have become a cutting-edge technology for the separation and enrichment of airborne fungal spores [20]. For example, Xu et al. [21] designed a microfluidic chip to extract airborne fungal spores with high precision. Yang et al. [22] proposed a method for capturing and detecting disease spores based on microfluidic chips and diffraction fingerprints. The combination of microfluidic chip and impedance analyzer has been an important means to detect airborne fungi spores.

Therefore, for timely detection of airborne crop fungal spores. In this study, a method based on microfluidic separation and enrichment and impedance characteristics was proposed to detect spores of crop fungal diseases. The microfluidic chip can filter most of the impurities compared with the spore trap, and it can separate and enrich the target particles. Compared with air and other methods, the concentration of spores in the enrichment zone of the microfluidic chip is higher and the impurities are less. As a non-invasive and label-free electrochemical method, impedance detection is very sensitive. The impedance analyzer is convenient to carry and has low environmental requirements. In this study, a microfluidic chip with tertiary structure was designed for the direct separation and enrichment of *Ustilaginoidea virens* spores, *Magnaporthe grisea* spores, and *Aspergillus niger* spores from the air. In the collection area of the microfluidic chip, an impedance analyzer was used to measure the impedance characteristics of the fungal spores, and the fungal spores were classified based on the impedance characteristics of the fungal spores. The results of this study can be used for the detection of airborne crop fungal spores, providing a basis for subsequent research on the detection of crop fungal spores.

## 2. Materials and Methods

### 2.1. Working Principle

**Figure 1:**
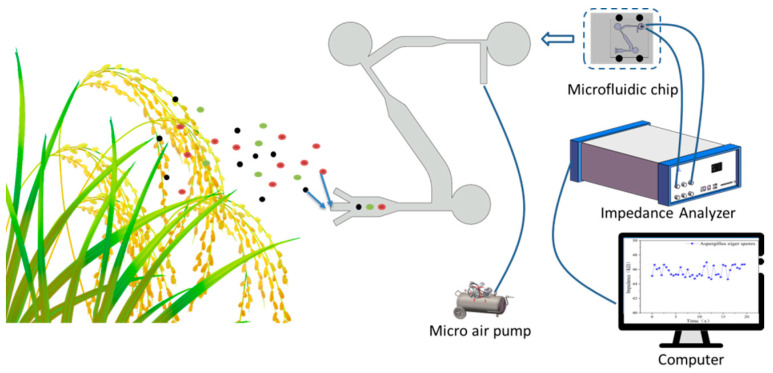
Schematic diagram of the working principle of the detection platform.

In order to realize the detection of crop fungal spores based on microfluidic separation and enrichment and AC impedance characteristics, the schematic diagram of the experimental platform of this study is shown in Figure 1. The platform consists of a microfluidic chip, an impedance analyzer, a micro air pump and a computer. The platform was placed in the field, and airborne fungal spores were collected by the microfluidic chip when crops are diseased. The fungal spores will be separated and enriched after entering the microfluidic chip, and the target fungal spores will enter the collection area. The concentration of fungal spores in the collection area will be much higher than the concentration of fungal spores in the air [23]. As shown in Figure 1, the microfluidic chip and the impedance analyzer were connected by a microfluidic chip fixture. The core function of the microfluidic chip fixture was the coupling between the electrode and the microfluidic chip, which was used to simplify the operation when the microfluidic chip was connected to the impedance analyzer, and to make the output data of the impedance analyzer more stable. The data were measured by impedance analyzer, and the four impedance characteristics of the fungal spore, i.e., impedance absolute value (abs), impedance real part (real), impedance imaginary part (imag), and impedance phase (phase), extracted. A classification model of *Ustilaginoidea virens* spores, *Magnaporthe grisea* spores, and *Aspergillus niger* spores was established to detect fungal spores.

### 2.2. Fungal Spores Sample Preparation

*Ustilaginoidea virens* spores and *Magnaporthe grisea* spores were provided by the China Rice Research Institute. For the convenience of experiments, *Ustilaginoidea virens* spores and *Magnaporthe grisea* spores were cultured on MS culture medium by liquid shaking, and spore suspensions were prepared. *Aspergillus niger spore* suspension was purchased from the Beijing Microbiological Culture Collection Center (BJMCC). In order to facilitate the design of microfluidic chip, the spores of fungal pathogens were analyzed by microscopy. The results showed that the spores of Ustilaginoidea virens were round and 3–5 µm in size, while the spores of *Aspergillus niger* were dark brown with conidiophore and enlarged apical sac, and the size was 3–5 µm. *Magnaporthe grisea* spores are ovular and 8–15 µm in size.

### 2.3. Theory and Working Principle for Microfluidic Chip

Airborne fungal spores are affected by the airflow in the microchannel of the microfluidic chip, some particles with sufficient momentum can rush into the enrichment area, and some particles with insufficient momentum will enter the next part of the channel with the deflection of the airflow. The behavior of such particles can be characterized by the Stokes number [24].

(1) $$stk=\frac{\rho_{p}d_{p}^{2}C_{c}V}{9\mu W}$$

where $d_{p}^{}$ is the particle size, m; $\rho_{p}$ is particle density, 1000 kg/m^−3^; μ is the air viscosity, 1.81 × 10^−5^ N·s·m^−2^; V is the air velocity at the inlet of the microfluidic device, m/s^−1^; W is the nozzle width, m;. stk is a dimensionless number, which describes the behavior of suspended particles in fluid. Particles with sufficient inertia will impact forward, while smaller particles with lower inertia will be suspended in the air flow. The diffusion and collision behaviors of particles are usually related to $Kn_{p}$. *Kn*_p_ is generally used to judge whether the fluid is suitable for the continuity hypothesis. *Kn*_p_ = 0.4–20 is considered the “transition zone” or “slip zone”, assuming that the particles are still moving in the continuous air flow. $C_{c}$ is the slip correction factor based on particle size, which is used to correct the difference from the actual value; it can be obtained from the following Formula (2) [24].

(2) $$C_{c}=1+0.5Kn_{p}\left[2.34+1.05\exp\left(-0.195Kn_{p}\right)\right]$$

where $Kn_{p}$ is the particle Knudsen number, defined as 2λ/$d_{p}^{}$, and λ is the mean free path of air molecules (m).

The enrichment efficiency of particles was calculated as the following Formula (3)

(3) $$\eta =\frac{N_{i}}{N_{j}}\times 100\%$$

where $N_{i}$ is the number of particles collected in the collection area, $N_{j}$ is the number of particles released, and η is the enrichment efficiency of the rich area.

### 2.4. Design of Microfluidic Chip

**Figure 2:**
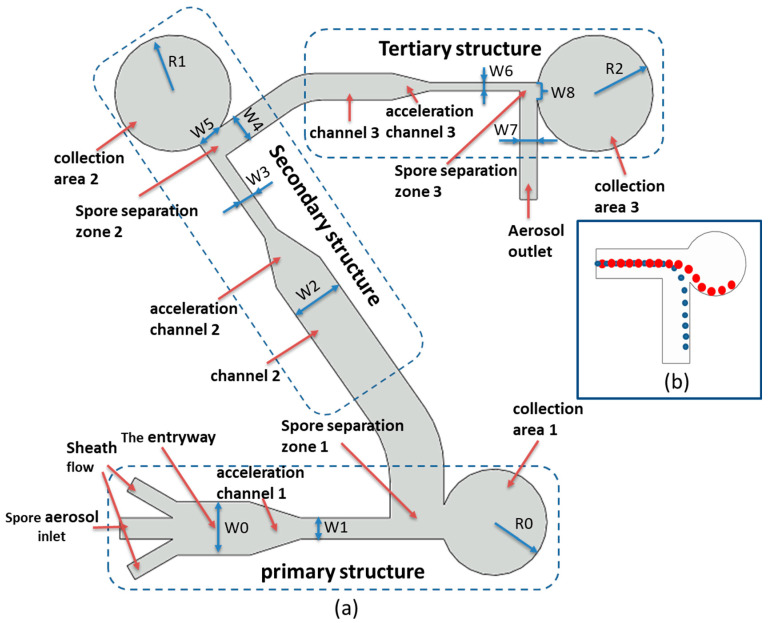
(**a**) Schematic diagram of the microfluidic chip structure. The chip consists of a tertiary structure. R0, R1, and R2 represent the radius of the first, second, and third enrichment regions, respectively, and W0–W7 represent the corresponding channel widths. (**b**) Sketch of the particle motion mechanism: small particles will follow the airflow into the next channel, and large particles will rush into the enrichment area. The red arrows point to the chip structure name, and the blue arrows indicate the chip size.

The schematic diagram of the microfluidic chip designed in this study is shown in Figure 2. The designed microfluidic chip consists of tertiary structures, each of which includes an acceleration channel, a fungal spore separation area and a fungal spore collection area. In order to better collect the target fungal spores, the inlet of the microfluidic chip was set as one fungal spore inlet and two aggregated sheath flows. Focused sheath flow ensures that fungal spore aerosols form a single aligned fungal spore airflow on the centerline. The acceleration channel can accelerate the fungal spores for the better separation of large particles of fungal spores.

The principle of the spore separation zone is shown in Figure 2b. When spore particles of different sizes pass through the acceleration channel, the larger spore particles will have larger inertia, and the smaller spore particles will obtain relatively small inertia. Therefore, larger spore particles will rush into the collection area, while smaller spore particles will enter the next channel.

### 2.5. Numerical Analysis

In this study, AutoCAD 2019 software was used to draw the structure diagram of the microfluidic chip, and then imported into COMSOL Multiphysics 5.5 software. Use COMSOL Multiphysics 5.5 Laminar Flow Module and Fluid Flow Particle Tracing Module to simulate the motion trajectories of particles in microfluidic channels and analyze the factors that affect particle motion behavior. During the simulation, the particle collision with the inner wall of the microfluidic chip is inelastic, and if the particle contacts the inner wall, the particles freeze permanently on the inner wall. The boundary conditions are set to fully develop the flow. The particle density was set to 1050 kg/m^3^. There were 100 spore particles evenly distributed at the spore aerosol inlet, and the inlet flow rate was 12.5 mL/min. The sheath flow inlet flow rate was 10 mL/min. The inlet width of the microfluidic chip was W0 = 4000 µm, the channel W1 = 2000 µm, the radius of the collection area 1 was R0 = 5000 µm, the width of the channel 2 was W2 = 5000 µm. The radius of the collection area 2 was equal to the radius of the collection area 3 R1 = R2 = 5500 µm. All microchannel heights were 100 µm. Collection area 1 collects particles larger than 15 μm (excluding 15 μm), collection area 2 collects particles of 5–15 μm (excluding 15 μm, including 5 μm), and collection area 3 collects particles of 3–5 μm (excluding 5 μm, including 3 μm). Collection area 2 can collect *Ustilaginoidea virens* spores and *Aspergillus niger* spores, and collection area 3 can collect *Magnaporthe grisea* spores. The values of W3/W4 and W3/W5 can determine the collection rate of collection area 2, and the values of W6/W7 and W6/W8 can determine the enrichment rate of collection area 3. Change the collection effect of collection areas 2 and 3 by changing W3/W4, W3/W5, W6/W7, and W6/W8 values (range 1.2–2.1). Select the optimal collection effect of collection area 2 and collection area 3, and design the microfluidic chip structure with the highest collection rate.

### 2.6. Detection Principle for Fungal Spores Based on Impedance Characteristics

Different types of fungal spores show different impedance characteristics due to their different sizes and shapes. Therefore, the impedance characteristics of fungal spores can be used to classify different types of fungal spores [16,25]. For a certain sample, the sample and detection properties have been determined, while the signal frequency needs to be optimized for sensitive and stable results. Inappropriate frequencies would lead to inaccurate results due to the electrode polarization effect and the electrical noise [14,26]. In order to select the frequency more intuitively, we introduce a parameter, C, which is defined as:

(4) $$C=|\frac{\Delta Z}{Z_{0}}|$$

(5) $$C_{1}=|\frac{\Delta Z_{1}}{Z_{0}}|$$

(6) $$C_{2}=|\frac{\Delta Z_{2}}{Z_{0}}|$$

where *Z*_0_ is the impedance of Magnaporthe grisea spores, *Z*_1_ is the im-pedance of Ustilaginoidea virens spores, and *Z*_2_ is the impedance of Aspergillus niger spores. Δ*Z* is the difference between the impedances of two different fungal spores, defined as Δ*Z*_1_ = *Z*_1_ − *Z*_0_, Δ*Z*_2_ = *Z*_2_ − *Z*_0_.

### 2.7. Evaluation Index for Fungal Spores Classification Result

In the field of machine learning, confusion matrix visualization tools, especially for supervised learning, are mainly used to compare classification results with actual measured values and can display the accuracy of classification results in a confusion matrix. Commonly used performance evaluation indicators for classification models include accuracy, precision, recall, and F1-Score. The calculation formula is shown in (7)–(10).

(7) $$\mathrm{Accuracy}=\frac{TP+TN}{TP+FP+TN+FN}$$

(8) $$\mathrm{Precision}=\frac{TP}{TP+FP}$$

(9) $$\mathrm{Recall}=\frac{TP}{TP+FN}$$

(10) $$\mathrm{F1-Score}=\frac{2\times \mathrm{Precision}\times \mathrm{Recall}}{\mathrm{Precision}+\mathrm{Recall}}$$

where TP (true positive) is used to represent the positive samples predicted by the model as positive; FP (false positive) is used to represent the negative samples predicted as positive by the model; FN (false negative) is used to represent the positive samples predicted as negative by the model; TN (true negative) is used to represent a negative sample predicted by the model to be negative.

## 3. Results and Discussion

### 3.1. Simulation Results of Microfluidic Chip

The channel width or width ratio of the microfluidic chip was changed to find the better effect of enriching particles in the collection area, to optimize the structure of the microfluidic chip to obtain a microfluidic chip with better functions. This section takes 10 µm spore particles and 4 µm spore particles as examples to study the relationship between channel width and enrichment effect.

**Figure 3:**
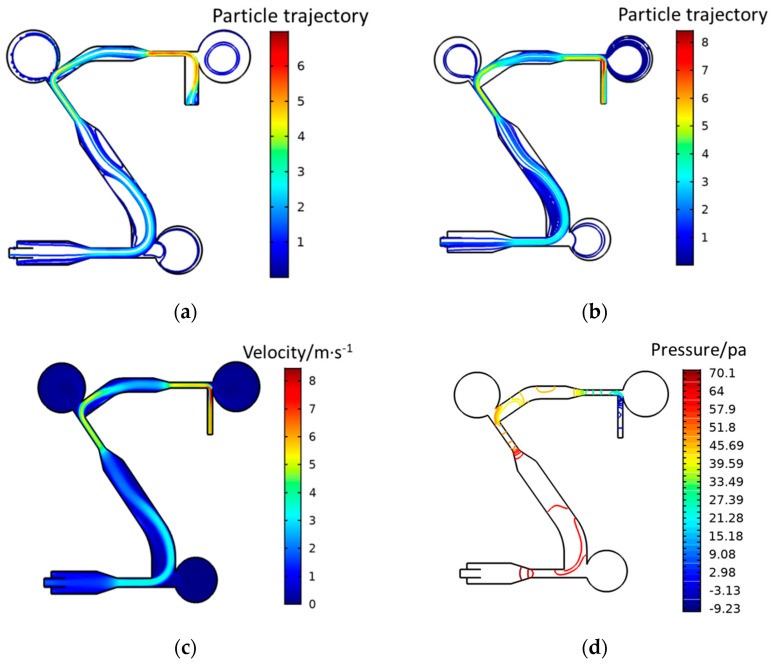
Simulation results of the microfluidic chip. (**a**) 4 µm particle trajectory, (**b**) 10 µm particle trajectory, (**c**) speed distribution, (**d**) pressure distribution.

Figure 3a,b represent the simulation result of the two kinds of particles, Figure 3c is the velocity distribution of the microchannel of the microfluidic chip, and Figure 3d is the pressure distribution intensity in the microfluidic chip. As shown in Figure 3, the aerosol enters the channel from the inlet and obtains an initial velocity of horizontal rightward, and the focusing sheath flow will focus the aerosol on the centerline. When the particles reach the first-stage spore separation area, the large-sized particles rush into the collection area due to their large inertia, and the small-sized particles will escape with the deflection of the airflow due to insufficient inertia. The velocity distribution and pressure distribution in the microfluidic chip play an important role in the collection of particles. Changing the width ratio of the channel can change the velocity distribution and pressure distribution in the microfluidic chip, thereby changing the particle enrichment efficiency of the microfluidic chip.

**Figure 4:**
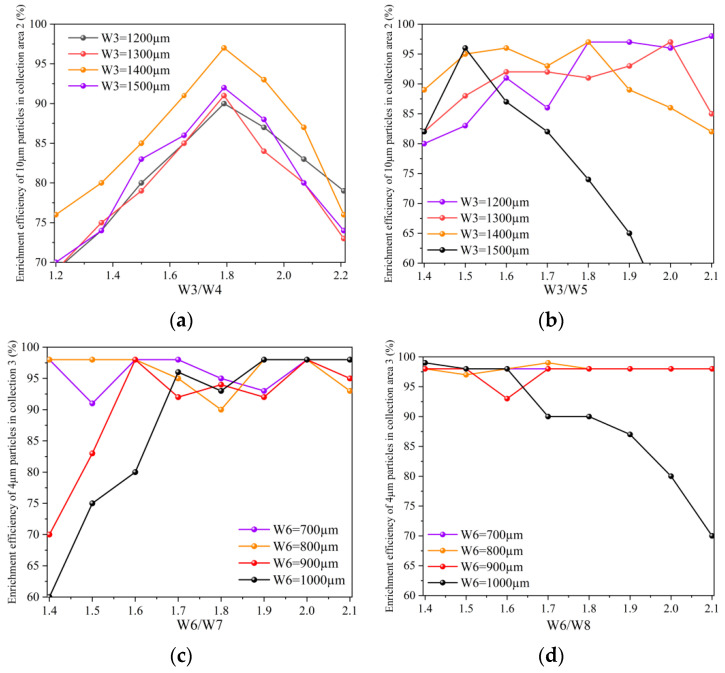
Chip simulation results (**a**) Collection efficiency corresponding to different W3/W4 ratios, (**b**) Collection efficiency corresponding to different W3/W5 ratios, (**c**) Collection efficiency corresponding to different W6/W7, (**d**) Collection efficiency corresponding to different W6/W8.

Figure 4a shows the relationship between the channel widths W3, W3/W4 and the enrichment efficiency of 10 µm particles. As can be seen from the Figure 4a, when W3 = 1400 µm and W3/W4 = 1.8, the separation and enrichment effect of 10 µm particles is better. The enrichment efficiency of collection zone 2 can reach 97, at this time W3 = 1400 µm, W4 = 2500 µm. Similarly, Figure 4b shows the relationship between the channel widths W3, W3/W5, and the enrichment efficiency of 10 µm particles. It can be seen from the Figure 4b that when W3 = 1400 µm and W3/W5 = 1.8 or W3 = 1200 µm and W3/W5 = 1.8, the separation and enrichment effect of 10 µm particles is better, and the enrichment efficiency of collection zone 2 can reach 97. Considering the actual situation of preparing microfluidic chips, W3 = 1400 µm and W5 = 2500 µm were finally selected in this study.

Figure 4c,d shows the relationship between the channel widths W6, W6/W7, W6/W8, and the enrichment efficiency of 4 µm particles, respectively. It can be seen from Figure 4c,d that when W6 = 700 µm or 800 µm, the enrichment efficiency of 4 µm is higher and more stable. If it is difficult to make a channel width that is thinner and not an integer, so choose W6 = 800 µm and W6/W7 = 2, W6/W8 = 2. At this time, the separation and enrichment effect of 4 µm particles is better, and in the collection area 3, the enrichment efficiency can reach 98. At this time, W6 = 800 µm, W7 = W8 = 1600 µm.

Thus, the final structure of the microfluidic chip can be determined. For collection area 2, choose W3 = 1400 µm, W4 = W5 = 2500 µm, and for collection area 3 choose W6 = 800 µm, W7 = W8 = 1600 µm. At this time, the microfluidic chip has a better separation and enrichment effect on the target particles. The enrichment efficiency of 10 µm particles in collection area 2 can reach 97, and the enrichment efficiency of 4 µm particles in collection area 3 can reach 98.

### 3.2. Test Results of Microfluidic Chip

#### 3.2.1. Fabrication of Microfluidic Chip

**Figure 5:**
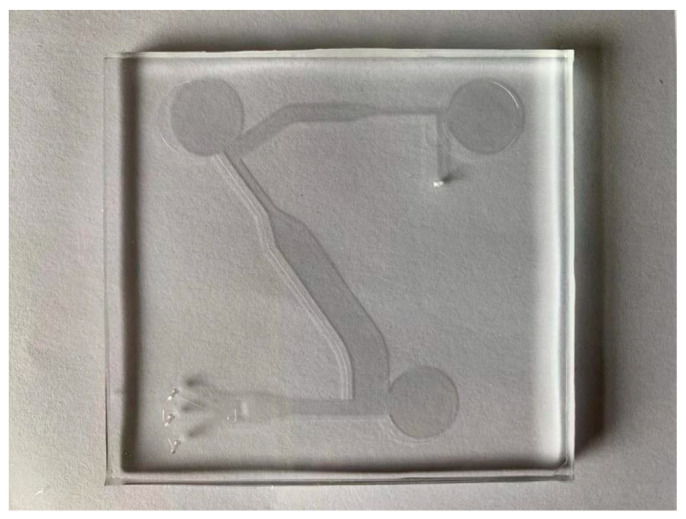
Microfluidic chip.

The microfluidic chip was fabricated by traditional photolithography, which was microfabricated with photoresist, mask, and ultraviolet light. The tools required for making are copper plate: scissors, tweezers, ruler, pipette gun, sandpaper, PDMS glue, and mold. First, Auto CAD 2019 was used to draw the microfluidic chip structure and make the mask. Secondly, a layer of 100 µm thin film is covered on the silicon wafer, and a layer of photoresist is uniformly covered on the surface of the film. Then, the microfluidic chip structure on the mask is imaged by exposure. Finally, polydimethylsiloxane (PDMS) was poured onto the mold and cured in an oven at 65 °C. Adhere the PDMS layer and substrate together. The fabricated microfluidic chip is shown in Figure 5.

#### 3.2.2. Test Platform

**Figure 6:**
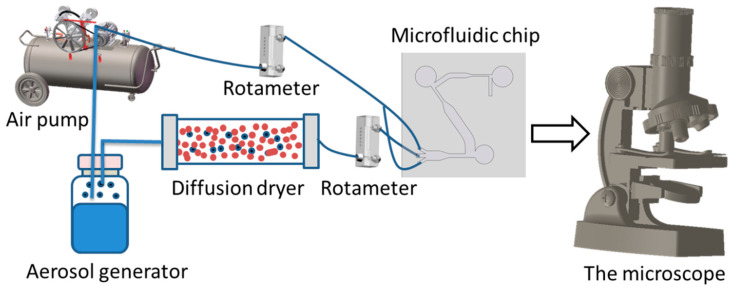
Schematic diagram of the microfluidic chip experimental setup.

Figure 6 shows a schematic diagram of the microfluidic chip experimental platform, which consists of an aerosol generator, a microfluidic chip, a microscope, an air pump, and two flow meters. Put the spore suspension into the aerosol generator, and the air compressor will send the compressed air into the aerosol generator for atomizing the spore aerosol. The dryer, after the aerosol generator, can remove the moisture in the aerosol. After that, the dry air flow will pass through the flowmeter (the flowmeter range is 6–60 mL·min^−1^), and the flowmeter will limit the flow rate of the flow to 12.5 mL·min^−1^ to obtain a stable air flow. Then, the airflow enters the microfluidic chip, and the spore aerosol is separated and enriched. The target spores will enter the collection area, so as to achieve the effect of enriching the spores. Finally, use a microscope to observe the collection of spores in the collection area of the microfluidic chip.

#### 3.2.3. Test Results of Microfluidic Chip Using Nanospheres

**Figure 7:**
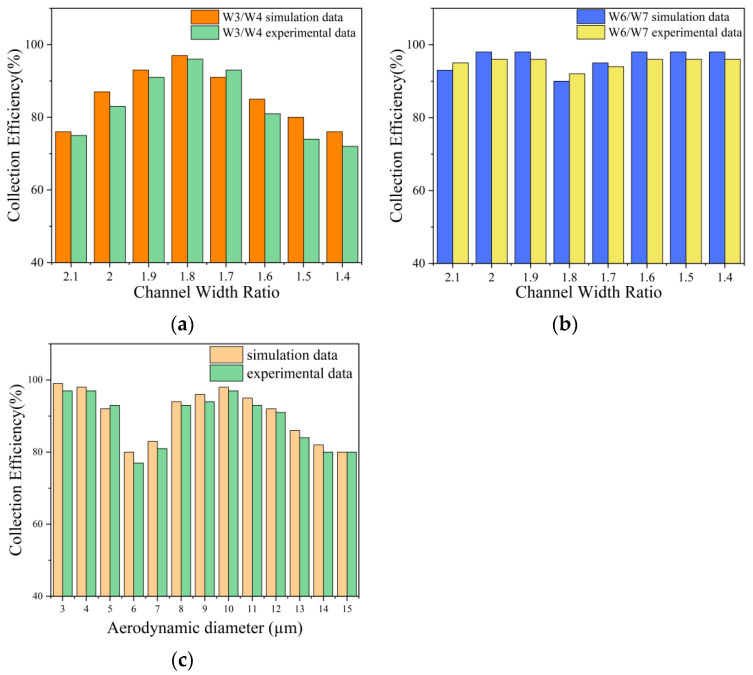
Comparison of chip test and simulation results. (**a**) Simulation and experimental results for W3/W4, (**b**) Simulation and experimental results for W6/W7, (**c**) is the collection effect of the microfluidic chip on the atomized particles of different sizes.

In order to verify the reliability of the simulation results of the microfluidic chip, this paper uses nanospheres to verify the enrichment efficiency of the microfluidic chip. The comparison between the experimental and simulation results of the microfluidic chip designed in this study is shown in Figure 7. During simulation, 3–15 µm particles were used in comsol software to calculate enrichment efficiency. In the experiment, 3–15 µm nanospheres were used to calculate the enrichment efficiency. There are many impurities in the spores and the size is uneven, so it is impossible to calculate the collection rate of each particle size. Nanospheres have no impurities and uniform size, so they are good experimental articles. Using nanospheres instead of spores, the collection efficiency of 3–15 μm particles by microfluidic chips can be calculated separately. The enrichment efficiency formula is Formula (3). Figure 7a,b shows the comparison diagrams of the simulation and experimental results of W3/W4 and W6/W7 collection efficiency. Figure 7c is the collection efficiency curve of atomized particles at each stage. The experimental results are slightly different from the simulation results. The experimental results show that the enrichment efficiency of 10 µm particles in collection zone 2 can reach 97, and the enrichment efficiency of 4 µm particles in collection zone 3 can reach 97.

It can be seen from Figure 7c that although the experimental results are slightly different from the simulation results, the particle loss rate is not high, and the microfluidic chip can be used to collect fungal spores in the air. The 6 µm, 7 µm, 14 µm, and 15 µm particles have lower collection rates relative to other particle sizes due to the loss of particles hitting the channel walls in the microfluidic chip. Particle loss mainly occurs in channel 2 of the second structure and channel 3 of the third structure, whereas 14 µm and 15 µm particles will collide with the channel wall in channel 2, and 6 µm and 7 µm will escape and collide with the channel wall in the spore separation zone 2 collision.

#### 3.2.4. Test Results of Microfluidic Chip Using Fungal Spores

**Figure 8:**
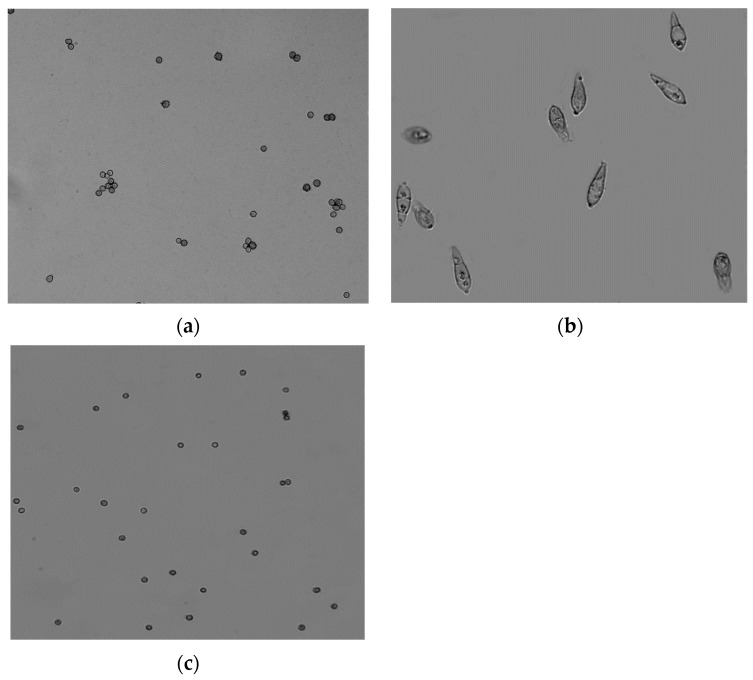
The images of spores in the enrichment area of the microfluidic chip, (**a**) is image of *Ustilaginoidea virens* spores, (**b**) is image of *Magnaporthe grisea* spores, (**c**) is image of *Aspergillus brasiliensis* spores.

Use the platform shown in Figure 6 to conduct the experiment. Put the spores into the aerosol generator and turn on the air pump. After the fungal spores were collected on the microfluidic chip, the PDMS layer of the microfluidic chip was removed in the laboratory to directly expose the microchannel and the collected bioaerosol, and the collected particles were observed by microscopy. Figure 8 shows the image of *Ustilaginoidea virens* spores, *Magnaporthe grisea* spores, and *Aspergillus niger* spores enriched in a chip taken using a microscope. It can be seen from the image that the spores can be enriched in the enrichment area, and the spores can be relatively evenly distributed in the enrichment area.

### 3.3. Detection and Classification Results Based on Fungal Spores Impedance Characteristics

#### 3.3.1. Results of Impedance Characteristics for Fungal Spores

**Figure 9:**
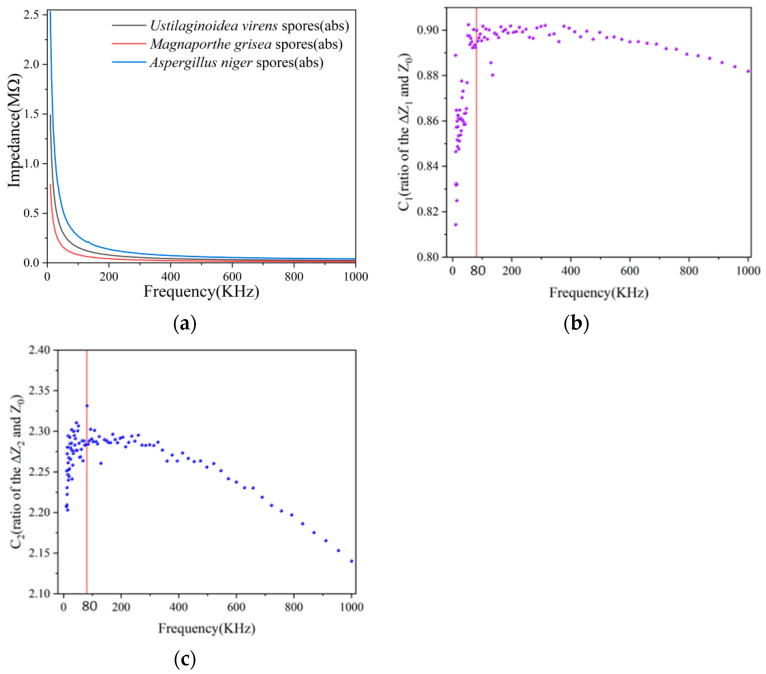
Choose the best measurement frequency, 80 KHz is more suitable (**a**) Impedance changes of three spores at 10–1000 KHz, (**b**) *C*_1_ value at different frequencies, (**c**) *C*_2_ value at different frequencies.

We measured Magnaporthe grisea spores, Ustilaginoidea virens spores, and Aspergillus Niger spores using an impedance analyzer. As shown in Figure 9a, we screened the impedance of the system, from 10 KHz to 1000 KHz. The impedance of the three fungal spores decreased with the increase of frequency. In order to make the difference in the electrical response between different fungal spores more obvious, a more suitable frequency needs to be selected, and experiments at a suitable frequency will obtain clear and stable results. In order to select the frequency more intuitively, we refer to Formulas (5) and (6). Figure 9b,c shows the values of *C*_1_ and *C*_2_ at each frequency, and it can be seen that *C*_1_ and *C*_2_ are not a constant. In the range of 10 KHz–1000 KHz, the phenomenon that the frequency is too low or too high will lead to lower detection sensitivity is consistent, which is consistent with the phenomenon in most articles [13,26,27]. At 80 KHz, the electrical impedance differences of the three fungal spores was more obvious and easier to distinguish. Therefore, in all the following experiments, the measurement frequency is chosen to be 80 KHz.

**Figure 10:**
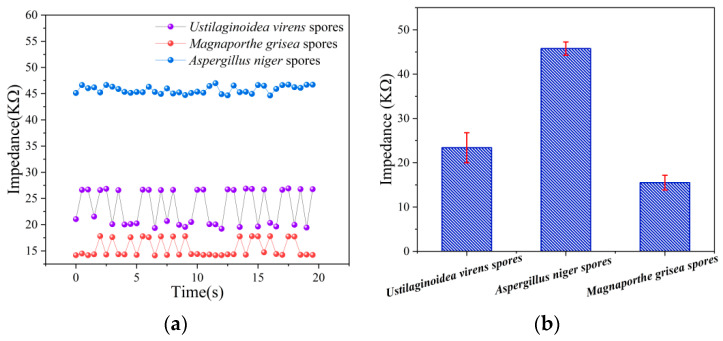
The curve of three kinds of spore impedance with time (**a**) Variation of impedance of three spores with time at 80 KHz (**b**) The impedance changes of the three spores at 80 KHz, the values shown represent the average of 10 experimental measurements for each spore.

*Ustilaginoidea virens fungal* spores, *Magnaporthe grisea fungal* spores, and *Aspergillus niger* fungal spores were detected at 80 KHz at 5 × 10^6^ CFU/mL. Figure 10a shows the change of impedance with time. The experiment was tested for 10 times, the mean and standard deviation were calculated, and the histogram of fungal spore impedance was drawn as shown in Figure 10b. In these 10 experiments, the average impedance range of *Ustilaginoidea virens* spores was 23.38 ± 3.39 MΩ, the average impedance range of *Aspergillus niger* spores was 45.76 ± 0.71 MΩ, and the average impedance range of *Magnaporthe grisea* spores was 15.5 ± 1.67 MΩ. Therefore, three types of spores can basically be distinguished using impedance data.

#### 3.3.2. Classification Result for Fungal Spores Based on Impedance Characteristics

**Figure 11:**
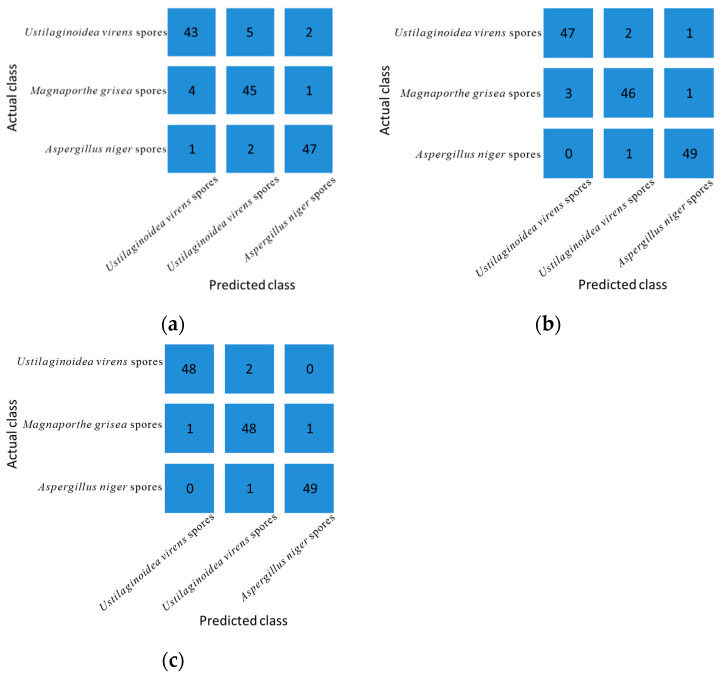
Confusion matrix for different classification models (**a**) KNN classification model confusion matrix (**b**) RF classification model confusion matrix (**c**) SVM classification model confusion matrix.

To further confirm the results reflected in Figure 10. In this study, a large number of experiments were conducted, and 300 sets of data were randomly selected to establish KNN, RF and SVM classification models. Three fungal spores were classified using KNN, RF and SVM models. K-nearest neighbor (KNN) algorithm is one of the most basic and simple machine learning algorithms. It can be used for both classification and regression. KNN classifies by measuring the distance between different eigenvalues. When using the KNN model for classification, 150 sets of data were used as the training set, and the K value of the KNN algorithm was estimated through 10 cross-validations. RF (random forest) generates multiple different data sets by sampling data sets, trains a classification tree on each data set, and finally combines the prediction results of each classification tree as the prediction results of random forests. When using the RF model for classification, the number of sub-datasets (n_esimators) determines the accuracy of the RF model. The selection range of n_esimators is set to 1~101, the step size is 10, and 10 times of cross-validation is used to select the optimal n_esimators. Finally select n_esimators as 61. SVM (Support Vector Machine) is a kind of generalized linear classifier that classifies data in a supervised learning way.The SVM algorithm has perfect mathematical theory. The SVM model uses a Gaussian kernel function. In this study, a confusion matrix was used to evaluate the classification model performance. The classification results are shown in Figure 11. Each row of the matrix represents the actual category of the sample, and each column represents the predicted category of the sample. Figure 11a is the result of the KNN classification model, Figure 11b is the result of the RF classification model, and Figure 11c is the result of the SVM classification model.

Table 1 shows the classification results of three fungal spores by KNN classification model, RF classification model and SVM classification model. Table 2 shows the accuracy, precision, recall and F1-Score of the three classification models. Table 3 shows the mean accuracy, mean precision, mean recall, and mean F1-Score of the three classification models.

It can be seen from Table 2 that the accuracy, precision, recall, and F1-Score of the SVM model for the classification results of the three fungal spores are higher than those of the KNN and RF models. The accuracy rate of SVM model for *Ustilaginoidea virens* spores is 98, the precision was 96, the recall rate was 97.96, and the F1-Score was 95.49. The accuracy rate of SVM model for *Magnaporthe grisea* spores was 96.67, the precision was 96, the recall rate was 94.12, and the F1-Score was 95.05. The accuracy of the SVM model for A. niger spores was 98.67, the precision was 98, the recall rate was 98, and the F1-Score was 98. It can be seen from Table 3 that the SVM model has better classification performance than the KNN model and the RF model. The F1-Score value of the SVM model was higher, with an average of 96.18, which was higher than that of the KNN model and the RF model, so the SVM model has a better classification effect.

## 4. Conclusions

In this study, in order to realize the rapid and high-precision detection of airborne fungal spores, a method for detecting airborne fungal spores using a microfluidic chip combined with an impedance analyzer was proposed. The detection platform built in this paper was used to measure the impedance changes caused by three airborne fungal spores, and a classification model was established. The experimental results show that the accuracy, precision, recall, and F1-Score of the SVM model are all higher than 97.78, 96.67, 96.69, and 96.18, respectively. The impedance detection method can realize the identification or classification of spores. Compared with the traditional detection method, the spore detection time is greatly shortened, and the accuracy is higher, which is conducive to the early prevention of diseases. This method provides a new idea for early disease prevention, and the real-time monitoring of spores by impedance detection method deserves further exploration.

## Figures and Tables

**Table 1:** Classification results of different classification models.

Species	Basic Indicators											
	KNN				RF				SVM			
	TP	TN	FP	FN	TP	TN	FP	FN	TP	TN	FP	FN
*Ustilaginoidea virens* spores	43	95	7	5	47	97	3	3	48	99	2	1
*Magnaporthe grisea* spores	45	93	5	7	46	97	4	3	48	97	2	3
*Aspergillus niger* spores	47	97	3	3	49	98	1	2	49	99	1	1

**Table 2:** Comparative analysis of different classification modes (%).

Species	Classification Results											
	Accuracy			Precision			Recall			F1-Score		
	KNN	RF	SVM	KNN	RF	SVM	KNN	RF	SVM	KNN	RF	SVM
*Ustilaginoidea virens* spores	92.00	96.00	98.00	86.00	94.00	96.00	89.59	94.00	97.96	87.76	94.00	96.49
*Magnaporthe grisea* spores	92.00	95.33	96.67	90.00	92.00	96.00	86.54	93.88	94.12	88.24	92.93	95.05
*Aspergillus niger* spores	96.00	98.00	98.67	94.00	98.00	98.00	94.00	96.08	98.00	94.00	97.03	98.00

**Table 3:** Comparison of the overall average recognition ability of different classification models (%).

Indexes	Classification Model		
	KNN	RF	SVM
Accuracy	93.33	96.44	97.78
Precision	90.00	94.67	96.67
Recall	90.04	94.65	96.69
F1-Score	90.00	94.65	96.18

## Data Availability

Data is contained within the article.
